# Virtual non-iodine photon-counting CT-angiography for aortic valve calcification scoring

**DOI:** 10.1038/s41598-024-54918-9

**Published:** 2024-02-27

**Authors:** Philipp Feldle, Marit Scheuber, Jan-Peter Grunz, Julius F. Heidenreich, Pauline Pannenbecker, Conrads Nora, Henner Huflage, Thorsten A. Bley, Bernhard Petritsch

**Affiliations:** 1 Department of Diagnostic and Interventional Radiology, University Hospital Würzburg, Oberdürrbacherstr. 6, 97080 Würzburg, Germany; 2 Department of Diagnostic and Interventional Radiology, Klinikum Klagenfurt am Wörthersee, Feschnigstr. 11, 9020 Klagenfurt am Wörthersee, Austria

**Keywords:** Computed tomography, Valvular disease

## Abstract

Photon-counting detector (PCD)-CT allows for reconstruction of virtual non-iodine (VNI) images from contrast-enhanced datasets. This study assesses the diagnostic performance of aortic valve calcification scoring (AVCS) derived from VNI datasets generated with a 1st generation clinical dual-source PCD-CT. AVCS was evaluated in 123 patients (statistical analysis only comprising patients with aortic valve calcifications [n = 56; 63.2 ± 11.6 years]), who underwent contrast enhanced electrocardiogram-gated (either prospective or retrospective or both) cardiac CT on a clinical PCD system. Patient data was reconstructed at 70 keV employing a VNI reconstruction algorithm. True non-contrast (TNC) scans at 70 keV without quantum iterative reconstruction served as reference in all individuals. Subgroup analysis was performed in 17 patients who received both, prospectively and retrospectively gated contrast enhanced scans (n = 8 with aortic valve calcifications). VNI images with prospective/retrospective gating had an overall sensitivity of 69.2%/56.0%, specificity of 100%/100%, accuracy of 85.4%/81.0%, positive predictive value of 100%/100%, and a negative predictive value of 78.2%/75.0%. VNI images with retrospective gating achieved similar results. For both gating approaches, AVCS_VNI_ showed high correlation (r = 0.983, *P* < 0.001 for prospective; r = 0.986, *P* < 0.001 for retrospective) with AVCS_TNC_. Subgroup analyses demonstrated excellent intra-individual correlation between different acquisition modes (r = 0.986, *P* < 0.001). Thus, VNI images derived from cardiac PCD-CT allow for excellent diagnostic performance in the assessment of AVCS, suggesting potential for the omission of true non-contrast scans in the clinical workup of patients with aortic calcifications.

## Introduction

Since the introduction of transcatheter aortic valve implantation (TAVI) in 2002, there has been tremendous advancement concerning not only the procedure itself, but also the associated imaging for treatment planning and device selection^1^. Today, computed tomography angiography (CTA) represents the established reference standard for evaluation of the access route, annular sizing, and risk determination for periprocedural annular injury^2,3^.

To date, additional non-contrast CT for aortic valve calcium quantification is not considered an essential portion of clinical routine imaging, but may have utility in a number of special settings and clinical scenarios^4^. Since aortic stenosis (AS) severity is known to correlate well with the aortic valve calcium load, CT-based calcium scoring can be problem solving when Doppler echo-cardiographic assessment of AS severity is hampered^5,6^. For instance, individuals with low-flow/low-gradient AS due to left ventricular dysfunction with limited left ventricular ejection fraction constitute one patient group benefiting from this scoring approach. Several studies have tried to find gender-specific aortic valve calcium score cut-off values that indicate the presence of high-grade AS^5–8^. In this context, an aortic valve calcification score (AVCS) ≥ 1300 in women or ≥ 2000 in men can be considered severe^4^. Moreover, aortic valve calcifications, when protruding into the lumen, are associated with increased risk of post-interventional complications, including para-valvular leakage, prosthesis dislodgement, obstruction of coronary ostia, calcific embolism/stroke and annular rupture, the latter being associated with particularly high mortality^9–12^. Nonetheless, both the SCCT (Society of Cardiovascular Computed Tomography) and ESCR (European Society of Cardiovascular Radiology) deem non-contrast CT with quantification of AVCS only necessary in selected patients prior to TAVI^2,3^. While this recommendation may be associated with increased examination complexity and a concomitant increase of radiation dose, it must be stated that the latter is modest and should not raise major concerns regarding the eligible population for TAVI.

Recently introduced photon-counting detectors (PCD) offer a wide spectrum of promising advantages in cardiovascular CT imaging when compared to conventional energy-integrating detector (EID)-based scanners^13–16^. PCD technology allows for energy discrimination at high temporal and spatial resolution by individually counting every incoming photon, and weighting it with regard to its transported energy^17,18^. Similar to dual-energy computed tomography approaches like dual-source, dual-layer or rapid kVp switching technology^19^, this offers possibilities for multi-material differentiation with the opportunity to create virtual non-iodine (VNI) images from CTA datasets^20,21^. In the context of pre-TAVI CT evaluation, this means that a true non-contrast (TNC) scan, which is normally required for aortic valve calcium scoring, can potentially be omitted, possibly diminishing both the cumulative radiation dose and examination time. Whereas VNI was predominantly proposed in the context of “classic” coronary artery calcium scoring in terms of a cardiovascular risk-stratification thus far^22,23^, a recent study by Mergen et al. demonstrated the potential of this technique for valvular calcium quantification from late iodine enhancement cardiac scans^24^.

Thus, the purpose of our study was to assess the diagnostic performance of first generation dual-source PCD-CT VNI imaging for quantification of aortic valve calcification, mass, and volume score from coronary CT angiographic (cCTA) images.

## Materials and methods

### Patient population and study design

**Figure 1:**
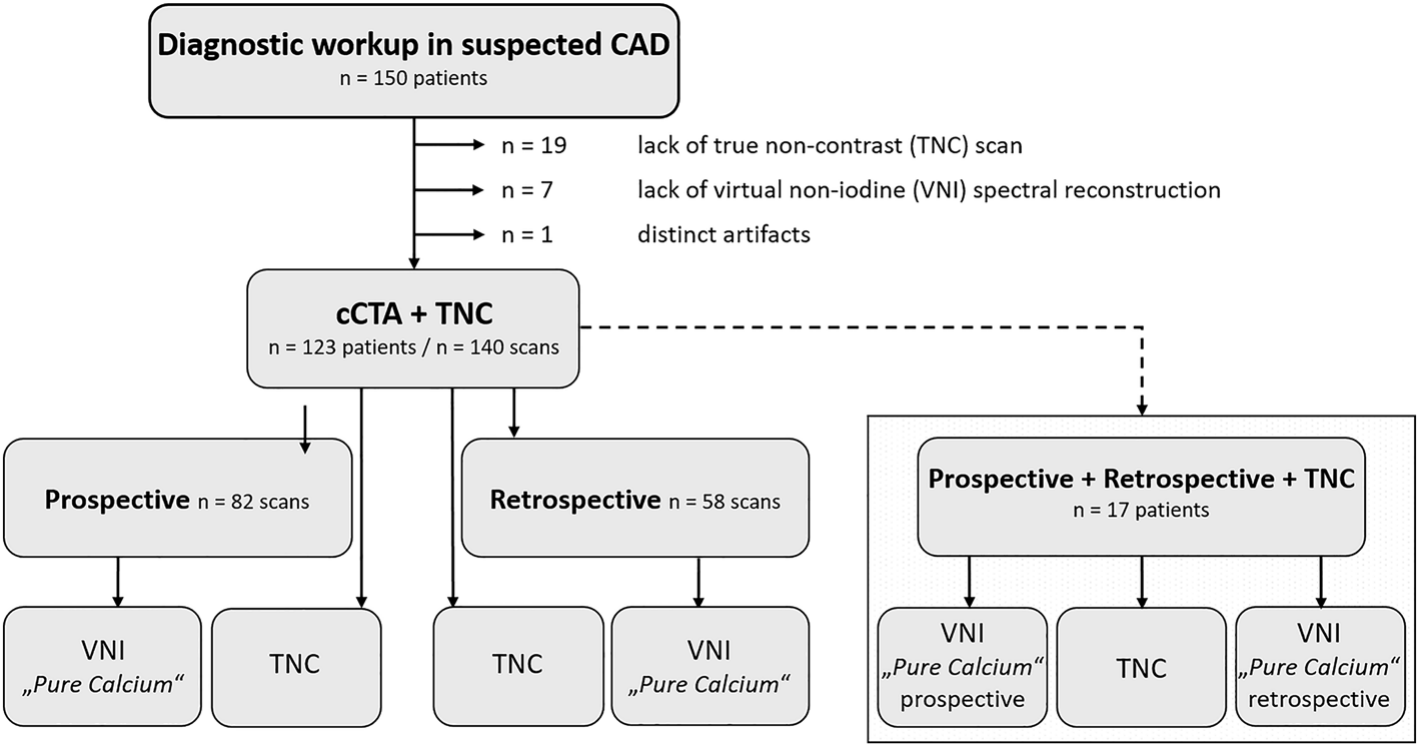
Flowchart shows selection of study population. Twenty-seven patients were excluded because of lacking a true non-contrast/virtual non-iodine scan or due to distinct artifacts. A total of 140 scans in 123 patients were available for final evaluation. *CAD—*Coronary artery disease*, cCTA*—coronary CT angiography*, TNC—*true non-contrast*, VNI*—virtual non-iodine.

The institutional review board of the University of Würzburg (Germany) approved the design of this retrospective single-center study and waived the need for additional written informed consent. The research was carried out in accordance with local legislation and the Declaration of Helsinki. From a total of 150 patients who underwent CT as part of routine diagnostic workup in suspected coronary artery disease (CAD) between November 2022 and June 2023, 123 patients (56 male, 67 female; mean age 63.2 ± 11.6 years) were included for final analysis. All patients received a TNC scan, which served as the reference standard for AVCS. Depending on the heart rate and rhythm, electrocardiogram-gated cCTA was either performed in a prospective (in form of an ultra-fast gated high-pitch “flash” scan; ≤ 62 bpm, rhythmic) or in a retrospective (≥ 63 bpm and/or arrhythmic) manner. In 17 patients of the prospective group, cCTA needed to be repeated as a retrospective acquisition, resulting in a subgroup featuring both, prospective and retrospective VNI datasets. Detailed information concerning the study design is presented in Fig. 1.

### CT scan acquisitions

All scans were acquired using a clinical 1st generation dual-source PCD-CT (Naeotom Alpha, Siemens Healthineers, Forchheim, Germany) equipped with two cadmium-telluride based photon-counting detectors (QuantaMax, Siemens Healthineers) and running the software version VA40. A collimation of 144 × 0.4 mm was employed for all examinations. First, unenhanced TNC data was acquired with a tube voltage of Sn 100 kVp (0.4 mm tin filter) and tube-current time product defined by an image quality index of 19. Of note, Apfaltrer et al. showed excellent agreement in calcium scoring protocols between 120 kVp and Sn 100 kVp despite significant radiation dose reduction^25^. Second, cCTA data was acquired with a tube voltage of 120 kVp in QuantumPlus mode and an IQ level of 64. Gantry rotation time was 0.25 s each. Automated tube-current modulation (CARE Dose4D, Siemens Healthineers) and tube-potential control (Care keV, Siemens Healthineers) were applied in all cCTA examinations. The same amount of contrast medium was administered in prospective and retrospective cCTA protocols. Detailed acquisition, radiation dose and contrast protocol parameters are provided in Table 1.

**Table 1:** Acquisition, radiation dose and reconstruction parameters of the true non-contrast group, prospective “Flash” cCTA and retrospective cCTA.

	TNC	Prosp. “Flash” cCTA	Retrosp. cCTA
Scan mode	Tin-filtered	QuantumPlus	QuantumPlus
Gating	Prospective	Prospective	Retrospective
Collimation	144 × 0.4	144 × 0.4	144 × 0.4
Rotation time [s]	0.25	0.25	0.25
Pitch	3.2	3.2	Variable*
Tube potential [kV]	Sn100	120	120
Image quality setting	IQ 19	IQ 64	IQ 64
Volume CT dose index [mGy]**	1.1	2.8	44.6
Dose length product [mGy cm]**	21.4	51.6	691.0
Automatic tube current modulation	On	On	On
Automatic tube potential control	On	On	On
Contrast medium [ml]	–	60	60
Flow rate [ml/s]	–	5.0	5.0
Iodine delivery rate [mg/s]	–	1750	1750
Trigger threshold; ascending aorta [HU]	–	100	100
FOV [mm]	160	160	160
Matrix size	512 × 512	512 × 512	512 × 512
Slice thickness [mm]	3	3	3
Increment	1.5	1.5	1.5
Kernel	Qr36	Qr36	Qr36
keV level for reconstruction	70	70	70

*cCTA* coronary CT angiography, *FOV* field of view, *HU* Hounsfield units, *IQ* image quality index, *Sn* tin-prefiltered, *TNC* True non-contrast; *depending on the heart rate, **provided as medians.

### Calcium scoring image reconstruction

The cCTA datasets were postprocessed by applying a VNI algorithm (PureCalcium, Siemens Healthineers) as previously described^20^. According to recent literature as well as the vendors recommendation, monoenergetic TNC reconstructions at 70 keV without quantum iterative reconstruction (QIR) served as the reference standard^22,23,26^. All images for calcium scoring were reconstructed with identical parameters (recommended by the vendor; factory protocol), which are summarized in Table 1.

### Image analysis

**Figure 2:**
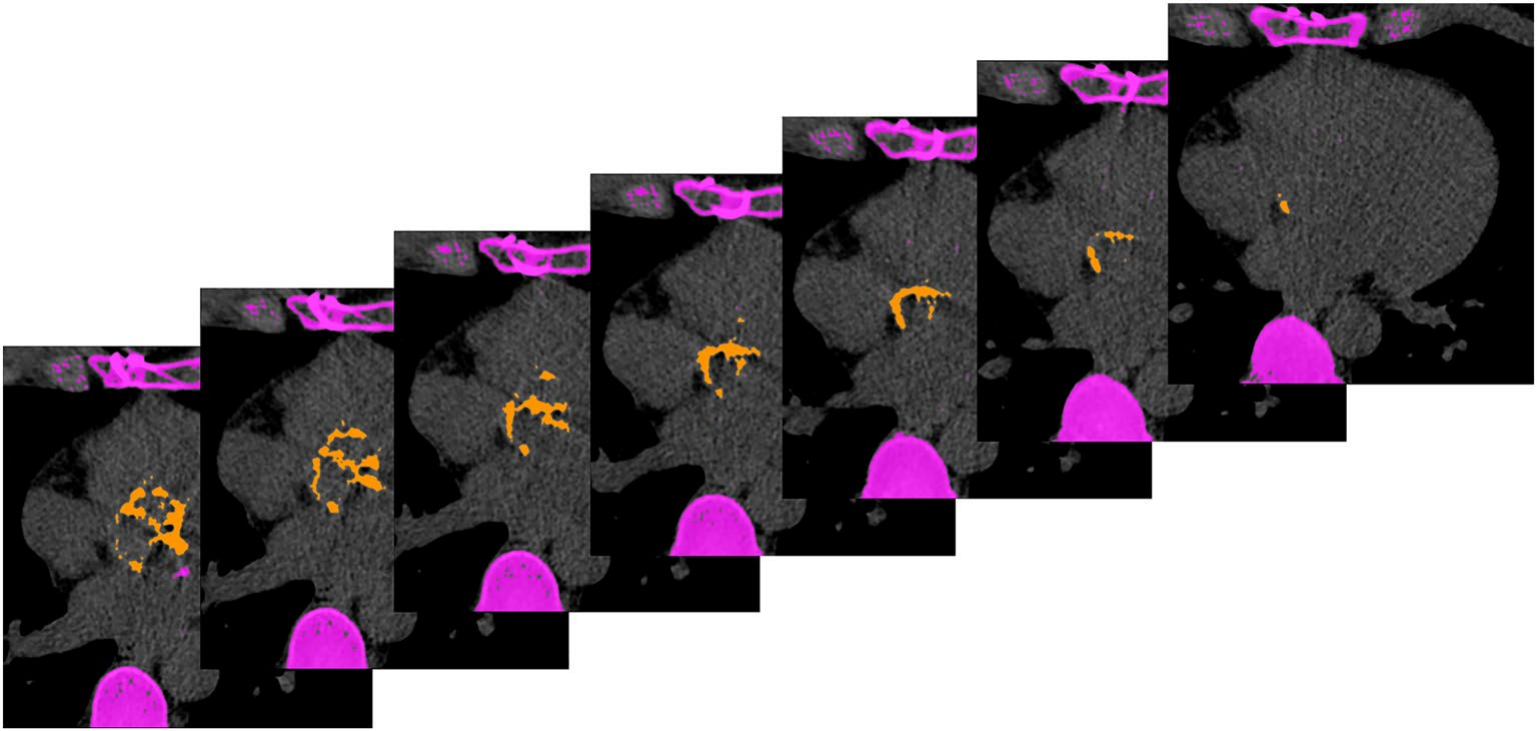
Segmented true non-contrast CT demonstrating severe aortic calcification in a male patient. The aortic valve calcification score (AVCS) was 5808 in this case.

Two radiologists (with 1 and 13 years of experience in cardiovascular imaging, respectively) evaluated all calcium-scoring CT datasets side by side in a random order for AVCS, using a commercially available software (syngo.via VB60, Siemens Healthineers). According to current consensus documents, only valvular calcifications were segmented, ensuring the exclusion of coronary, left ventricular outflow tract, and aortic wall calcifications^2,4^. Figure 2 shows an example of how aortic valve calcium segmentation was performed. For every dataset, the Agatston score, aortic valve calcification mass and volume were evaluated.

### Statistical analysis

Shapiro–Wilk tests were conducted to assess the normal distribution of metrically-scaled variables. If normally distributed, items are presented as mean ± standard deviation, otherwise as median with interquartile range. Classification functions of diagnostic accuracy were calculated separately for VNI images derived from prospective “flash” and retrospective spiral acquisitions. Pearson's correlation coefficient was computed to assess the relationship between measurements in true non-contrast and virtual non-iodine datasets. *P* values ≤ 0.05 were considered to indicate the significance of test results. Analyses were performed employing dedicated statistical software (SPSS 28, IBM, Armonk, USA).

## Results

### Aortic valve calcium scoring

In 67 patients, no aortic valve calcifications were ascertained (AVCS_TNC_ of 0). In the remaining 56 patients, which were enrolled for statistical calculations, the median AVCS_TNC_, mass_TNC_, and volume scores were 119 (54–603), 20 mg (9–121 mg), and 111 mm^3^ (52–495 mm^3^), respectively. Six male individuals displayed an AVCS_TNC_ > 2000, while no female individuals exceeded an AVCS_TNC_ > 1300.

For both cCTA based approaches, AVCS_VNI_ showed high correlation with AVCS_TNC_ (*r* = 0.983, *P* < 0.001 for prospective; *r* = 0.986, *P* < 0.001 for retrospective). Similarly, mass_VNI_ and volume_VNI_ score each showed high correlation with mass_TNC_ and volume_TNC_ (mass: *r* = 0.997, *P* < 0.001 for prospective; *r* = 0.988, *P* < 0.001 for retrospective/volume: *r* = 0.982, *P* < 0.001 for prospective; *r* = 0.982, *P* < 0.001 for retrospective).

**Figure 3:**
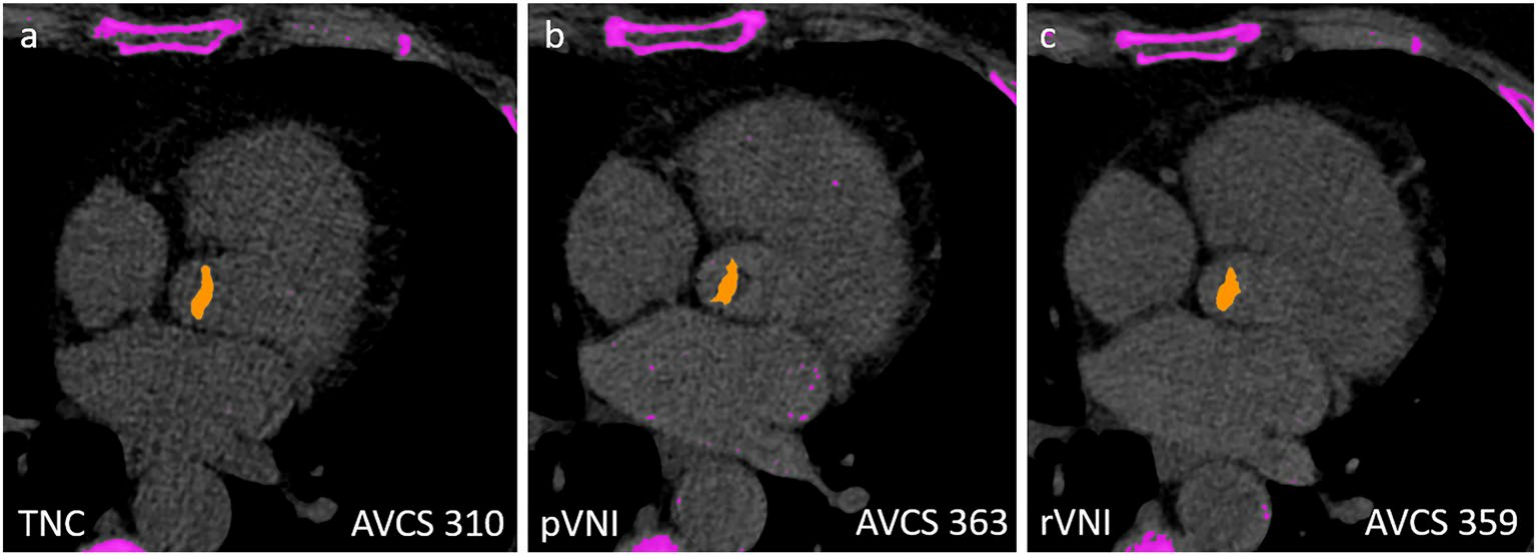
True non-contrast (TNC) CT (**a**), prospective virtual non-iodine (pVNI) CT (**b**), and retrospective virtual non-iodine (rVNI) CT (**c**) in a 62-year old woman demonstrate high concordance of aortic valve calcification score (AVCS) across all three calcium-scoring approaches.

Prospective cCTA-based VNI images had sensitivity of 69.2% (95% confidence interval: 52.4%, 83.0%), specificity of 100% (91.8%, 100%), accuracy of 85.4% (75.8%, 92.2%), positive predictive value of 100% (87.2%, 100%), and a negative predictive value of 78.2% (69.1%, 85.1%) for the assessment of aortic valve calcifications (Fig. 3). Retrospective cCTA-based VNI images showed nearly identical results, demonstrating an overall sensitivity of 56.0% (34.9%, 75.6%), specificity of 100% (89.4%, 100%), accuracy of 81.0% (68.6%, 90.1%), positive predictive value of 100% (76.8%, 100%), and a negative predictive value of 75.0% (65.8%, 82.4%), for assessment of aortic valve calcifications (Fig. 3).

**Figure 4:**
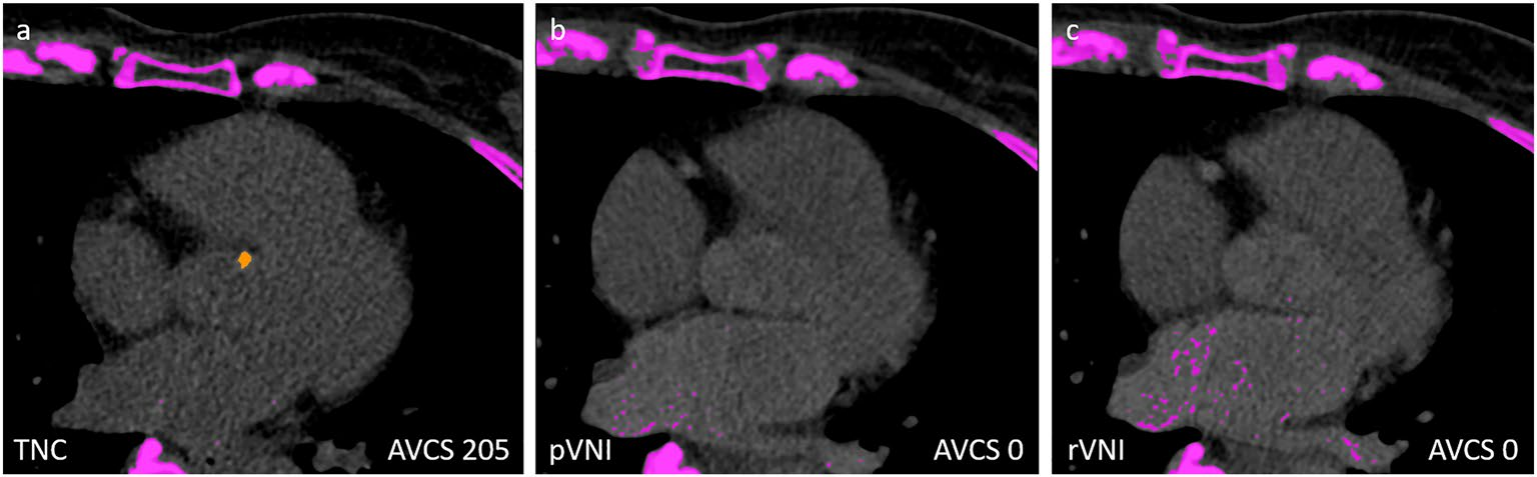
True non-contrast (TNC) CT (**a**), prospective virtual non-iodine (pVNI) CT (**b**), and retrospective virtual non-iodine (rVNI) CT (**c**) in a 75-year old woman from show false negative aortic valve calcification score (AVCS) in both VNI approaches.

With regard to the established AVCS cut-off values (Agatston score > 1300 for women and > 2000 for men suggesting the presence of severe AS), in only one out of 56 patients with aortic valve calcifications, stenosis estimation changed due to incorrectly low AVCS in retrospective VNI images (AVCS_TNC_ 2227 *vs*. AVCS_VNI_ 1808). However, in a total of 15 patients, all of which presented with very low AVCS_TNC_ < 60 [median 51; range 4–60], AVCS_VNI_ scores were false negative (Fig. 4).

### Subgroup analysis

In the subgroup of 17 patients who received both cCTA acquisition protocols, 9 individuals did not exhibit any aortic valve calcifications in either TNC or VNI images (AVCS of 0). In the remaining 8 patients, which were enrolled for statistical calculations, the median AVCS_TNC_, mass_TNC_, and volume_TNC_ scores were 112 (46–160), 18 mg (7–27 mg), and 107 mm^3^ (46–138 mm^3^), respectively.

In the intra-individual comparison, both cCTA based approaches showed high correlation of AVCS (*r* = 0.986, *P* < 0.001), mass (*r* = 0.992, *P* < 0.001), and volume (*r* = 0.987, *P* < 0.001) scores.

## Discussion

This retrospective study on 123 patients demonstrated that virtual non-iodine postprocessing of spectral dual-source photon-counting detector CT data is a reliable technique for aortic valve calcification scoring. Iodine from contrast-enhanced scans can be digitally subtracted, allowing for aortic valve calcifications to be assessed and analyzed quantitatively without the necessity of acquiring a true non-contrast scan. For both prospective and retrospective gating-approaches, quantitative analyses of virtual non-iodine data provide high diagnostic accuracy and excellent correlation to true non-contrast imaging.

Previous studies have demonstrated the capability of PCD-CT to perform accurate coronary calcium scoring at various tube voltages, simultaneously offering potential for substantial radiation dose reduction when compared to conventional EID-CT^26–29^. Moreover, due to its intrinsic spectral resolution, PCD-CT offers the possibility to calculate virtual iodine-free images and thus render a separate scan for calcium scoring obsolete. Several investigations have evaluated the potential of PCD-CT derived VNI reconstructions for coronary artery calcium assessment and also for mitral annular and aortic valve calcification scoring^20,22–24^. Emrich et al. found, that the vendor-specific non-iodine algorithm named *PureCalcium* (used in the present study) outperforms standard virtual non-contrast reconstructions from spectral data^20^.

A comparatively low IQ level of 19 was applied for the TNC scan constituting the reference standard for AVCS analysis (compared to an IQ index of 64 in cCTA acquisitions), as higher IQ levels did not improve scoring accuracy in previous studies^29^. All reconstructions were computed at 70 keV. This keV level has been reported to show the least deviation of calcium scores derived from standard EID-CT and has therefore been established as diagnostic standard in PCD-CT-based calcium scoring^28,29^.

With one exception^24^, VNI technique was yet typically evaluated in the context of “classic” coronary artery calcium scoring as proposed by the 2019 American College of Cardiology/American Heart Association *Guideline on Primary Prevention of Cardiovascular Disease*^30^. Recently Mergen et al. showed that VNI created from cardiac late iodine enhancements scans offer accurate quantification, not only of coronary calcifications, but also of mitral annular and aortic valve calcifications^24^. However, there were some differences to our study setting. In addition to different software versions being used (VA40 *vs*. VA 50), we analyzed cCTA scans instead of late enhancement cardiac scans as “source data” for VNI generation. The lower attenuation of the blood pool on late iodine enhancement images might contribute to a more precise material decomposition, however, from a practical point of view, cCTA is more common in clinical routine, especially in the context of pre-TAVI evaluation were AVCS usually becomes relevant. It is worth mentioning that, in the study by Mergen et al., VNI reconstructions at 80 keV provided the best accuracy in AVCS, while reconstructions at other keV levels (e.g., 70 keV as used in our cohort) resulted in a significant underestimation of calcification score.

By allowing AVCS based on contrast-enhanced CT scans, concerns about prolonged examination protocols and higher radiation dose associated with additional TNC scans can be overcome. According to the ALARA—*as low as reasonable achievable*—principle, the positive side effect of dose saving is generally welcome, although one should not overestimate the relevance of radiation exposure in the aging population for which AV calcium quantification is usually considered, namely patients selected for TAVI procedures^2,3^. More important is the fact that up to 40% of patients show a discordant assessment of AS severity with echocardiography as the current reference standard test. Suchlike inaccuracies are commonly observed in patients with a severely decreased aortic valve area (≤ 1 cm^2^) but flow parameters only suggesting moderate disease (mean gradient < 40 mm Hg; peak flow < 4 m/s)^31^. The crucial advantage of performing AVCS assessment in this scenario is its independence of the hemodynamic situation. In summary, we do believe that VNI reconstructions could further strengthen the clinical acceptance of AVCS as a marker of stenosis severity, progression of valvular disease, and powerful predictor of adverse events^4^.

In our patient sample, we observed a relatively high number of false negative analyses (n = 15), resulting in a comparatively low negative predictive value. This observation is in line with findings from previous investigations, and most likely due to a combination of limited detectability of low-density calcifications, the reconstruction algorithm, and partial volume effects^20,32^. In this context, it should be emphasized that all individuals with false negative assessment in VNI datasets presented with a very low AVCS_TNC_ < 60. Due to the fact that only Agatston scores markedly > 1000 are considered relevant for classification of severe AS^4^, the clinical impact of this finding is presumably rather low. However, in individual cases, this inaccuracy could possibly lead to incorrect grading of AS. On the other hand, a high positive predictive value of both prospective and retrospective cCTA-based synthetic non-contrast images (both 100%), emphasizes the potential role of VNI images as a diagnostic tool for validation of echo-based diagnosis of severe AS or as a superior alternative in challenging clinical scenarios, like low-flow/low-gradient AS with limited left ventricular ejection fraction.

## Limitations

This study has several limitations that need to be addressed. First, due to dose saving reasons, our clinical CAD protocol features a tin-filtered 100 kVp scan for TNC calcium scoring instead of a 120 kVp filtered back-projection protocol, generally considered as standard of reference when calcium quantification is demanded. However, it has recently been shown that 70 keV reconstructions derived from Sn 100 kVp acquisitions allow for accurate calcium score calculation when compared to 120 kVp EID-CT scans^28^. Second, in the present CAD collective, only few patients presented with very high aortic valve calcium loads (only 6 male patients exceeded an AVCS > 2000). Third, according to the vendor, newer software versions (syngo.via VA50) provide improved spectral image quality and material decomposition compared to the software version we used in the present study. Additional analyses in a TAVI-specific cohort with expectably higher AVCS in the majority of patients should be performed in future studies. Third, the impact of aortic valve sclerosis characteristics, such as plaque size and density on AVCS_VNI_, mass_VNI_, and volume_VNI_ were not evaluated.

## Conclusion

VNI images derived from PCD-cCTA showed excellent diagnostic performance for assessing AVCS. Displaying potential for TAVI-associated risk assessment, aortic valve stenosis grading, as well as radiation dose reduction in clinical routine, VNI-based AVCS analysis may render a separate TNC scan unnecessary in the future.

## Data Availability

The datasets generated and/or analyzed during this study are not publicly available as CT data and DICOM headers contain patient information. Data can be obtained on reasonable request from the corresponding author.
